# Workplace Productivity Loss in Patients with Progressive Pulmonary Fibrosis: Data from the ILD-PRO Registry

**DOI:** 10.1007/s00408-026-00895-x

**Published:** 2026-05-27

**Authors:** Sharashchandra Shetty, Aparna C. Swaminathan, Peide Li, Megan L. Neely, Amy L Olson, Laurie D. Snyder, the ILD-PRO Registry investigators, Albert Baker, Debabrata Bandyopadhyay, Scott Beegle, John A. Belperio, Domingo Chardon, Rany Condos, Daniel Dilling, Bradford Bemiss, John Fitzgerald, Kevin R. Flaherty, Reginald Fowler, Mridu Gulati, Nishant Gupta, Amy Hajari Case, Mark Hamblin, David Hotchkin, Robert J. Kaner, Jad Kebbe, Hyun J. Kim, Ena Gupta, Justin Hewlett, Joseph A. Lasky, Hector Sanchez, Victor Pinto-Plata, Randolph Lipchik, LJason Lobo, Tracy R. Luckhardt, Yolanda Mageto, Toby M. Maher, Lake Morrison, Andrew Namen, Tessy Paul, Mary Porteous, Rishi Raj, Murali Ramaswamy, Jesse Roman, Ivan Rosas, Tonya Russell, Zeenat Safdar, Gabrielle Liu, Brian Southern, Mary E. Strek, Srihari Veeraraghavan, Rajat Walia, Timothy P. M. Whelan

**Affiliations:** 1 Boehringer Ingelheim Pharmaceuticals, Inc, 900 Ridgebury Rd, Ridgefield, CT 06877 USA; 2 Duke Clinical Research Institute, Durham, NC USA; 3 Duke University Medical Center, Durham, NC USA

**Keywords:** Absenteeism, Cost of illness, Interstitial lung diseases, Observational study, Presenteeism

## Abstract

**Purpose:**

Pulmonary fibrosis may impair an individual’s ability to work. We assessed the extent, cost and factors associated with workplace productivity loss among patients with progressive pulmonary fibrosis (PPF).

**Methods:**

The multi-center ILD-PRO Registry enrolled US patients with progressive interstitial lung diseases (ILDs) other than idiopathic pulmonary fibrosis. Workplace productivity loss was assessed using the Work Productivity and Activity Impairment (WPAI) questionnaire. Associations between patient characteristics and workplace productivity loss among employed patients were analyzed using logistic regression.

**Results:**

Among 175 employed patients of 597 who completed the WPAI questionnaire, 58.3% were female, median age was 58 years, median forced vital capacity (FVC) was 58.7% predicted; 20.0% used oxygen with activity and at rest. In the past 7 days, mean (SD) hours worked was 32.9 (15.8) and mean (SD) working hours lost was 10 (12). Mean (SD) percentage of working hours lost was 24.4 (30.2). Mean (SD) working hours lost due to absenteeism and presenteeism were 4 (9) and 8 (11), respectively. Among patients with any productivity loss, the estimated mean (SD) annual cost of productivity loss was $24,815 ($17,917). Lower FVC % predicted, higher (worse) St. George’s Respiratory Questionnaire scores and lower (worse) Cough and Sputum Assessment Questionnaire cough impact and symptoms scores were associated with greater odds of productivity loss.

**Conclusions:**

Patients with PPF showed significant impairment in workplace productivity. Worse lung function and worse scores on patient-reported outcomes were associated with a greater likelihood of productivity loss.

*Trial Registration* ClinicalTrials.gov; No: NCT01915511; registered August 5, 2013; URL: www.clinicaltrials.gov.

**Supplementary Information:**

The online version contains supplementary material available at 10.1007/s00408-026-00895-x.

## Introduction

Worsening lung fibrosis associated with an interstitial lung disease (ILD) other than idiopathic pulmonary fibrosis (IPF) is referred to as progressive pulmonary fibrosis (PPF) [1]. Patients with fibrosing ILDs often experience symptoms such as dyspnea, cough and fatigue, which impair quality of life and worsen as the disease progresses [2–5]. Anxiety and depression are also common in patients with ILDs, particularly in those with more advanced disease [6, 7]. ILD and its associated symptoms have been shown to have a deleterious impact on a patient’s ability to engage in work [8–10], but the impact of PPF on work productivity is not well understood. We used data from the ILD-PRO Registry [11] to assess workplace productivity loss, its cost, and factors associated with workplace productivity loss, among patients with PPF.

## Methods

### The ILD-PRO Registry

The ILD-PRO Registry enrolled patients aged ≥ 30 years with an ILD other than IPF that was diagnosed or confirmed at the enrolling center  [11]. Eligible patients had reticular abnormality and traction bronchiectasis (with or without honeycombing) on a high-resolution computed tomography (HRCT) scan and/or lung biopsy and met at least one of the following criteria for ILD progression within the prior 24 months: relative decline in forced vital capacity (FVC) % predicted ≥ 10%; relative decline in diffusing capacity of the lungs for carbon monoxide (DLco) % predicted ≥ 10%; relative decline in FVC % predicted ≥ 5–<10% plus worsened respiratory symptoms and/or increased extent of fibrotic changes on HRCT; worsened respiratory symptoms plus increased extent of fibrotic changes on HRCT. At enrollment, retrospective data were collected from electronic medical records. Patients were then followed prospectively while receiving usual care. In addition, a call center contacted participants every 6 months to confirm vital status and interactions with healthcare systems. The study was approved by the Duke University Institutional Review Board (Pro00046131). The protocol was approved by the relevant Institutional Review Boards and/or local Independent Ethics Committees prior to patient enrolment. All patients provided written informed consent. Data for these analyses were extracted from the database in May 2025.

## Workplace Productivity

Workplace productivity was assessed using the Work Productivity and Activity Impairment (WPAI) questionnaire (GH V2.0) [12, 13]. This questionnaire comprises six questions: one about whether the individual is employed and five about the effect of health problems on an individual’s ability to work and perform regular activities, with a recall period of 7 days (Table 1). The questionnaire was administered via the call center and was included in interviews from 16 August 2023 to 30 November 2024. Each participant only completed the questionnaire once.

**Table 1:** Responses to WPAI questionnaire among employed patients (*n* = 175)

1	Are you currently employed (working for pay)?	175 (100)
2	How many hours did you miss from work because of your health problems? *Include hours you missed on sick days*,* times you went in late*,* left early*, etc.,* because of your health problems*	3.6 (9.5)
3	How many hours did you miss from work because of any other reason, such as vacation, holidays, time off to participate in this study?	2.3 (6.5)
4	How many hours did you actually work?	32.9 (15.8)
5	How much did your health problems affect your productivity while you were working? *	2.4 (2.7)
6	How much did your health problems affect your ability to do your regular daily activities, other than work at a job?^†^	3.3 (2.9)

Data are n (%) or mean (SD). Questions two to six cover the previous 7 days

*Scored from 0 to 10, where 0 indicates that “health problems had no effect on my work” and 10 indicates that “health problems completely prevented me from working”. Nine patients had missing data on this question

^†^Scored from 0 to 10, where 0 indicates that “health problems had no effect on my daily activities” and 10 indicates that “health problems completely prevented me from doing my daily activities”

## Analyses

The characteristics of patients who completed the WPAI questionnaire were analyzed descriptively. Age and payor information were based on data collected at enrollment. Other variables were based on values from the clinical encounter prior to administration of the WPAI questionnaire. Absenteeism was defined as the number of hours missed from work because of health problems (based on question two). Presenteeism was calculated as the number of hours worked (based on question four) multiplied by the impact of health problems on work productivity on a scale of 0 (score of 0 on question five, *i.e*. “health problems had no effect on my work”) to 0.1 (score of 10 on question five, i.e. health problems completely prevented me from working”). Total workplace productivity loss was the total of absenteeism and presenteeism. Among employed patients, the annual cost of workplace productivity loss was estimated based on the median usual weekly earnings of full-time workers by age and sex provided by the US Bureau of Labor Statistics [14].

Associations between the following patient characteristics and any workplace productivity loss (loss > 0 vs. 0) were analyzed using logistic regression: age, sex, FVC % predicted, DLco % predicted, Cough and Sputum Assessment Questionnaire (CASA-Q) cough impact and cough symptoms domain scores [15], St. George’s Respiratory Questionnaire (SGRQ) total and domain scores [16], GAP index [17] and use of supplemental oxygen. Higher SGRQ total and domain scores indicate worse impairment. Lower CASA-Q scores indicate worse cough. The odds ratios describing the associations between SGRQ total and domain scores and CASA-Q domain scores and productivity loss were scaled using published estimates for minimal clinically important differences [18, 19]. Use of supplemental oxygen was assessed as a categorical variable (no use, use at rest, use at rest and with activity). Correlations between patient characteristics and workplace productivity loss (hours/week) were assessed using Spearman’s rank correlation coefficients (rho). Missing data were not imputed.

## Results

### Patients

Of 1000 patients enrolled into the ILD-PRO Registry, 597 patients (59.7%) completed the WPAI questionnaire. The characteristics at enrollment of patients who completed the WPAI questionnaire, patients who had not completed the questionnaire but were alive at the time the data were extracted from the database (*n* = 242) and patients who had died (*n* = 161) were similar (Table S1). Median (Q1, Q3) time from enrollment in the registry to completion of the WPAI questionnaire was 13.4 (5.8, 29.2) months. Of the patients who completed the questionnaire, 175 (29.3%) were employed. Among employed patients, median age was 58 years; 58.3% were female (Table 2). Median FVC was 58.7% predicted and DLco was 37.2% predicted; 20.0% were using oxygen with activity and at rest. Compared with unemployed patients, employed patients were younger (median 58 vs. 70 years), less likely to have smoked (5.1% vs. 12.3%), more likely to have private insurance (77.7% vs. 45.3%) and had better HRQL based on SGRQ scores (median SGRQ total score 41.2 vs. 48.2) (Table 2).

**Table 2:** Characteristics of patients who completed the WPAI questionnaire

	Employed patients (*n* = 175)		Unemployed patients (*n* = 422)	
	Summary measure	Missing data	Summary measure	Missing data
Female	102 (58.3)	–	265 (62.8)	–
Age, years	58 (48, 65)	–	70 (64, 75)	–
Body mass index, kg/m^2^	24.8 (21.4, 28.0)	5 (2.9)	23.6 (20.5, 27.0)	7 (1.7)
Race		10 (5.7)		13 (3.1)
White	115 (65.7)		326 (77.3)	
Black/African-American	41 (23.4)		60 (14.2)	
Asian	2 (1.1)		8 (1.9)	
Other	7 (4.0)		15 (3.6)	
Current/former smoker	9 (5.1)	–	52 (12.3)	–
Private insurance	136 (77.7)	3 (1.7)	191 (45.3)	6 (1.4)
ILD diagnoses		–		–
Autoimmune ILDs	115 (65.7)		251 (59.5)	
Hypersensitivity pneumonitis	21 (12.0)		76 (18.0)	
Idiopathic non-specific interstitial pneumonia	18 (10.3)		38 (9.0)	
Unclassifiable ILD	10 (5.7)		32 (7.6)	
Other ILDs	11 (6.3)		25 (5.9)	
FVC % predicted	58.7 (47.7, 73.0)	13 (7.4)	64.9 (50.6, 77.6)	22 (5.2)
DLco % predicted	37.2 (28.9, 51.4)	5 (2.9)	36.9 (28.1, 48.6)	15 (3.6)
GAP index	3 (2, 4)	13 (7.4)	4 (3, 5)	28 (6.6)
Using nintedanib	49 (28.0)	–	128 (30.3)	1 (0.2)
Using pirfenidone	3 (1.7)	–	20 (4.7)	1 (0.2)
Using immunosuppressant	137 (78.3)	–	296 (70.1)	1 (0.2)
CASA-Q cough impact domain score	73.4 (50.0, 96.9)	1 (0.6)	75.0 (53.1, 93.8)	10 (2.4)
CASA-Q cough symptoms domain score	58.3 (41.7, 83.3)	1 (0.6)	58.3 (41.7, 75.0)	9 (2.1)
SGRQ total score	41.2 (27.5, 56.8)	–	48.2 (33.6, 63.8)	17 (4.0)
SGRQ symptoms score	46.1 (25.4, 65.5)	–	50.6 (30.5, 65.9)	10 (2.4)
SGRQ activity score	59.5 (41.7, 79.2)	–	67.9 (53.5, 85.8)	12 (2.8)
SGRQ impact score	27.4 (15.1, 43.8)	–	33.7 (18.6, 53.4)	11 (2.6)

Data are median (Q1, Q3) or n (%) of patients

Age and payor information were assessed at enrollment. Other variables were assessed at the clinical encounter prior to administration of the WPAI questionnaire

CASA-Q, Cough and Sputum Assessment Questionnaire. DLco, diffusing capacity of the lungs for carbon monoxide. FVC, forced vital capacity. GAP, gender, age, physiology. SGRQ, St. George’s Respiratory Questionnaire

### Workplace Productivity Loss

The responses of the employed patients to each question in the WPAI questionnaire are summarized in Table 1. In the past 7 days, the mean (SD) total number of hours worked was 32.9 (15.8). When asked to score the impact of health problems on their workplace productivity from 0 to 10, where 0 indicated “health problems had no effect on my work” and 10 indicated “health problems completely prevented me from working”, the mean (SD) score was 2.4 (2.7). Workplace productivity loss is summarized in Table 3. Among patients with any productivity loss, the mean percentage of hours lost in the past 7 days was 24.4%. Among patients with presenteeism, the mean (SD) hours lost due to presenteeism was 8 (11). Among patients with absenteeism, the mean (SD) hours lost due to absenteeism was 4 (9). The mean number of hours lost and the mean percentage of hours lost were similar between male and female patients (Table 3). Among patients with any productivity loss, the estimated mean (SD) annual cost of productivity loss was $24,815 ($17,917).

**Table 3:** Workplace productivity loss among employed patients

	Males (*n* = 73)	Females (*n* = 102)	Total (*n* = 175)
Total hours worked	33 (16)	33 (16)	33 (16)
Hours lost			
Total	10 (10)	10 (13)	10 (12)
Absenteeism	5 (11)	3 (9)	4 (9)
Presenteeism	7 (8)	8 (12)	8 (11)
Percent of hours lost			
Total	24.3 (26.0)	24.5 (33.0)	24.4 (30.2)
Absenteeism	11.6 (26.4)	7.1 (21.4)	9.0 (23.6)
Presenteeism	17.8 (20.8)	20.1 (30.2)	19.1 (26.6)
Estimated annual cost of productivity loss, $			
Total	26,729 (16,797)	23,338 (18,748)	24,815 (17,917)
Absenteeism	31,180 (24,219)	22,195 (20,058)	26,809 (22,450)
Presenteeism	21,000 (13,707)	19,913 (18,154)	20,377 (16,332)

Data are mean (SD). Mean hours lost and mean estimated annual cost of productivity loss for absenteeism, presenteeism and any productivity loss were calculated among patients with absenteeism, presenteeism and any productivity loss, respectively

## Associations Between Patient Characteristics and Workplace Productivity Loss

**Fig. 1:**
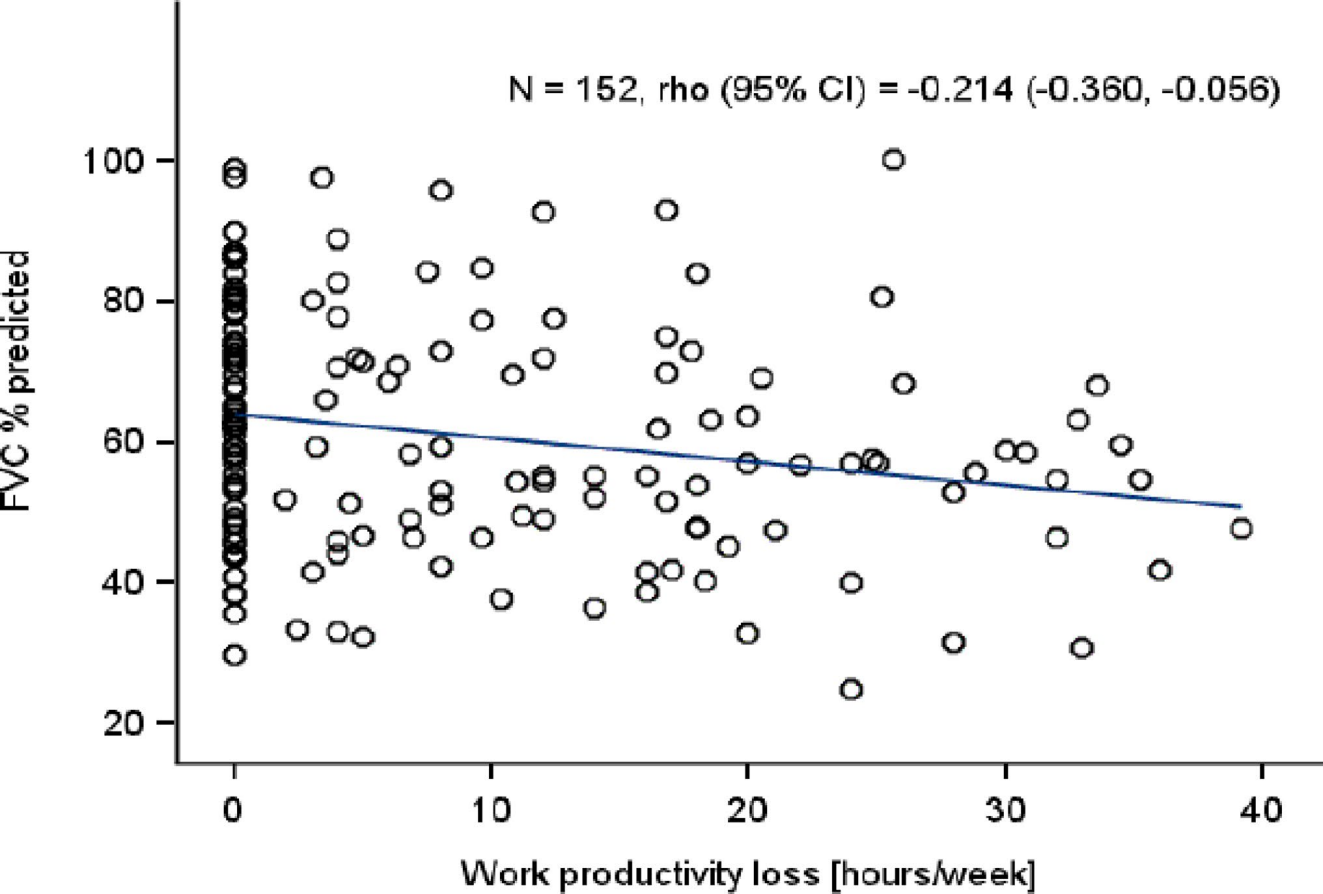
Correlation (rho) between FVC % predicted and workplace productivity loss

**Fig. 2:**
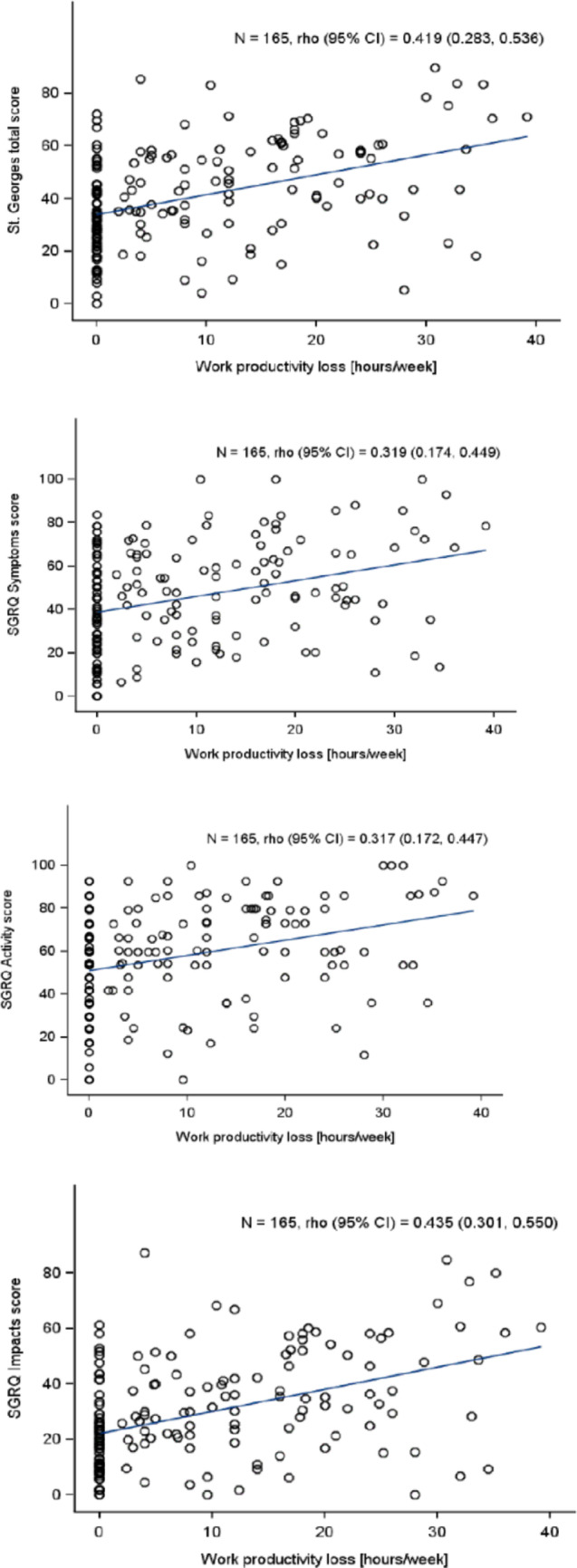
Correlation (rho) between SGRQ total, symptoms, activity and impact scores, and workplace productivity loss

**Fig. 3:**
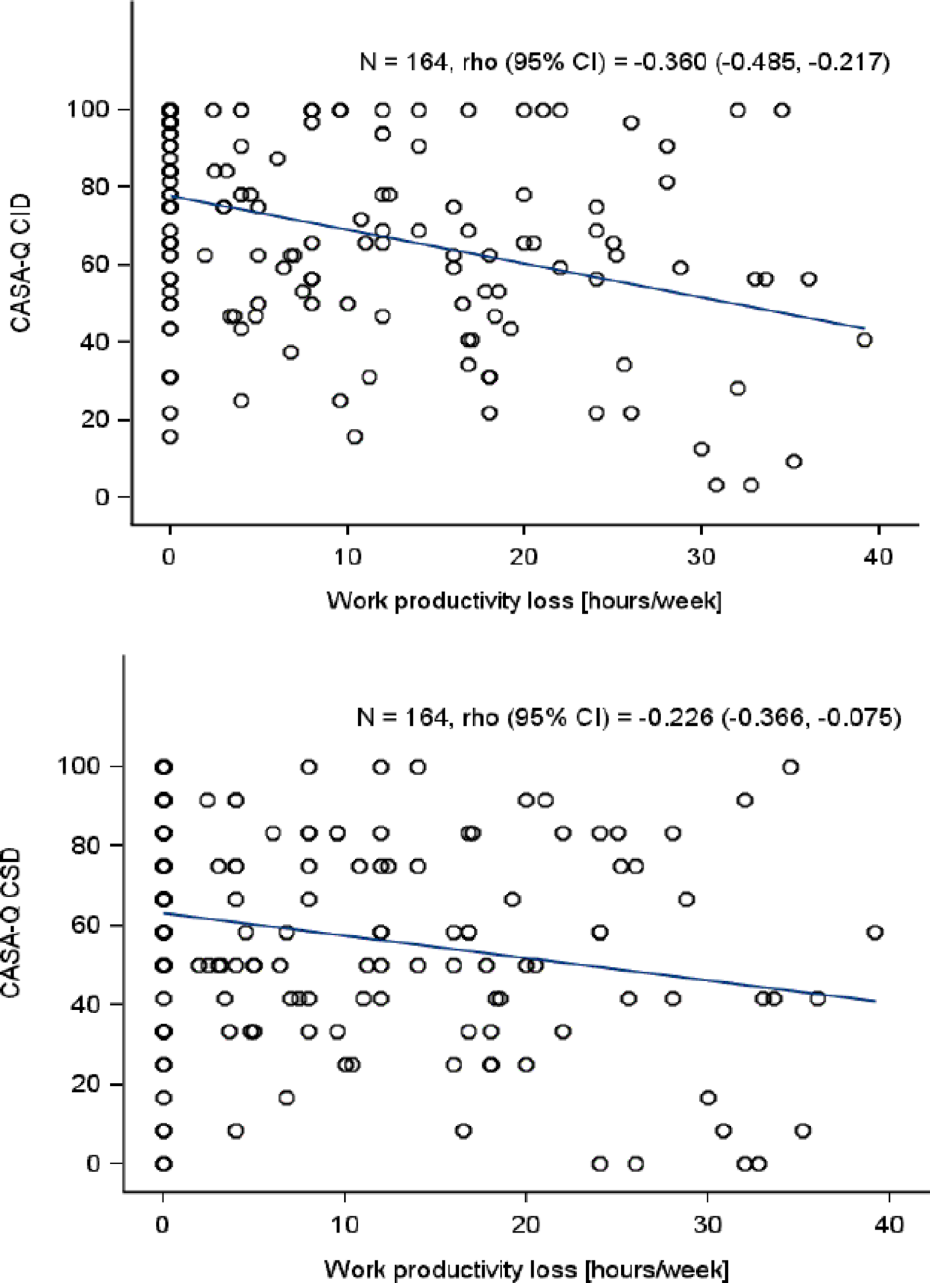
Correlation (rho) between CASA-Q cough impact domain and cough symptoms domain scores, and workplace productivity loss

Among employed patients who completed the WPAI questionnaire, the median (Q1, Q3) times from last assessment to completion of the WPAI questionnaire were 95 (55, 194) days for FVC, 125 (62, 244) days for DLco, 161 (79, 212) days for the CASA-Q and 162 (84, 212) days for the SGRQ. Lower FVC % predicted, higher (worse) SGRQ scores and lower (worse) CASA-Q domain scores were associated with greater odds of any workplace productivity loss (Table 4). Correlations (rho) between these characteristics and workplace productivity loss (hours/week) were − 0.21 for FVC % predicted (Fig. 1); 0.42, 0.32, 0.32 and 0.44 for the SGRQ total, symptoms, activity and impact scores, respectively (Fig. 2); and − 0.36 and − 0.23 for the CASA-Q cough impact and cough symptoms scores, respectively (Fig. 3). There were no significant associations between age, sex, DLco % predicted, or GAP index and the odds of any workplace productivity loss (Table 4). Correlations between these characteristics and workplace productivity loss (hours/week) are shown in Figures S1–S4.

**Table 4:** Associations between patient characteristics and any workplace productivity loss

Age, per 1 year higher	*N* included in the model	Odds ratio (95% CI)	*P*-value
	175	1.00 (0.97, 1.02)	0.74
Female sex	175	0.81 (0.43, 1.53)	0.52
FVC % predicted, per 1 unit higher	162	0.98 (0.96, 1.00)	0.039
DLco % predicted, per 1 unit higher	170	0.99 (0.97, 1.01)	0.13
CASA-Q cough impact domain score, per 11 points higher	174	0.75 (0.64, 0.87)	< 0.001
CASA-Q cough symptoms domain score, per 11 points higher	174	0.86 (0.75, 0.98)	0.026
SGRQ total score, per 7 points higher	175	1.37 (1.20, 1.58)	< 0.001
SGRQ symptoms score, per 8 points higher	175	1.24 (1.10, 1.39)	< 0.001
SGRQ activity score, per 5 points higher	175	1.14 (1.06, 1.22)	< 0.001
SGRQ impact score, per 7 points higher	175	1.41 (1.22, 1.64)	< 0.001
GAP index	162	1.12 (0.90, 1.39)	0.31

Lower CASA-Q scores indicate worse cough. Higher SGRQ scores indicate worse impairment

CASA-Q and SGRQ scores were scaled using estimates for minimal clinically important differences (18,19)

CASA-Q, Cough and Sputum Assessment Questionnaire. DLco, diffusing capacity of the lungs for carbon monoxide. FVC, forced vital capacity. GAP, gender, age, physiology. SGRQ, St George’s Respiratory Questionnaire

Excludes one patient with workplace productivity loss > 80 h/week

FVC, forced vital capacity

Excludes one patient with workplace productivity loss > 80 h/week

SGRQ, St. George’s Respiratory Questionnaire

Excludes one patient with workplace productivity loss > 80 h/week

CASA-Q, Cough and Sputum Assessment Questionnaire

## Discussion

These analyses assessed the extent and cost of workplace productivity loss, and the factors that may contribute to it, among patients with PPF participating in the ILD-PRO Registry. Only 29% of the 597 patients in the registry who completed the WPAI questionnaire were employed. Of the employed patients, among whom the median age was 58 years, 24% of hours were lost, with an estimated cost of productivity loss of $24,815 per individual per year.

In our study, twice as many hours work were lost due to presenteeism than absenteeism. Similarly, an analysis of data from 148 employed patients with various ILDs (including IPF) enrolled in the Canadian Registry for Pulmonary Fibrosis (CARE-PF) showed that mean productivity loss was 5.5 h per week due to presenteeism and 2.3 h per week due to absenteeism [8]. These findings are important, as presenteeism is generally under-recognized in evaluations of the cost of illness [20]. They suggest that strategies targeting presenteeism, such as optimizing the workplace environment or modifying job descriptions for patients with health issues, may be particularly important in minimizing the impact of lung fibrosis on workplace productivity.

While some patients with ILD have reduced workplace productivity, others are unable to work at all. Using a Delphi method, a panel of physicians who treated patients with ILDs believed that approximately 27% of patients with PPF retire early, 48% have total permanent disability and 23% lose their job due to their disease [21]. Data from an economic model estimating the burden of PPF in Europe suggested that among 86,794 individuals, there were 3,952,604 annual sick days associated with PPF, 23,174 early retirements, 41,748 permanently disabled patients and 19,789 job losses [22]. The annual loss of income due to sick days, early retirements, and disability was €1433 million [22].

Loss of workplace productivity adds to the economic burden of pulmonary fibrosis that also includes the costs of hospitalizations, tests used in diagnosis and monitoring, and medications [23–25]. A reduced ability to work, with resultant loss of earnings, has been reported as a cause of concern by patients with pulmonary fibrosis [26–28]. In a survey of 45 European patients with IPF, approximately one fifth of patients reported that an inability to work and resulting reduction in income caused stress and concern that they were an increasing burden to their families [27].

Strategies that help to preserve lung function or alleviate symptoms, as part of a holistic approach to the care of patients with pulmonary fibrosis [29, 30], might help improve patients’ ability to work. In our study, worse FVC % predicted and worse severity and impact of respiratory symptoms were significantly associated with workplace productivity loss. Similarly, in an analysis of 148 employed patients from CARE-PF, worse dyspnea and cough (measured using the University of California San Diego Shortness of Breath Questionnaire and a visual analog scale for severity of cough) were associated with loss of productivity [8]. However, among 113 employed patients with systemic autoimmune rheumatic disease-associated ILDs in CARE-PF, productivity loss was not associated with the severity of respiratory symptoms [9]. It is possible that unmeasured factors related to the impact of the systemic disease might have contributed to productivity loss in these patients. Oxygen use is an indicator of severe disease and places practical limitations on patients [31, 32]. In our study, we were unable to assess the likelihood of workplace productivity loss based on use of oxygen. There were more patients using supplemental oxygen at rest and with activity or only with activity than only at rest. This led to an odds ratio that was unrealistically large when use only at rest was compared with use at rest and with activity.

Our analyses have limitations. The ILD-PRO Registry enrolled patients at specialist centers, potentially limiting the generalizability of the results to the wider community of patients with PPF. The relevance of the findings to the broader population of patients with ILDs that are not progressive is unknown. Not all patients participating in the ILD-PRO Registry completed the WPAI questionnaire. The WPAI questionnaire does not capture reasons for productivity loss or potential confounders such as type of employment and pay. Only the impact of ILD on employed patients was assessed; we did not ascertain whether patients who were not employed had lost their job or stopped working due to ILD.

## Conclusions

Patients with PPF in the ILD-PRO Registry showed significant impairment in workplace productivity. Worse lung function and worse scores on patient-reported outcomes were associated with a greater likelihood of workplace productivity loss. These findings suggest that measures to preserve lung function and quality of life in patients with ILD might help to retain patients’ ability to work.

## Supplementary Information

Below is the link to the electronic supplementary material.

Supplementary Material 1

## Data Availability

The datasets analyzed during the current study are not publicly available, but they are available from the corresponding author on reasonable request.

## References

[1] Raghu G, Remy-Jardin M, Richeldi L, Thomson CC, Inoue Y, Johkoh T, Kreuter M, Lynch DA, Maher TM, Martinez FJ, Molina-Molina M, Myers JL, Nicholson AG, Ryerson CJ, Strek ME, Troy LK, Wijsenbeek M, Mammen MJ, Hossain T, Bissell BD, Herman DD, Hon SM, Kheir F, Khor YH, Macrea M, Antoniou KM, Bouros D, Buendia-Roldan I, Caro F, Crestani B, Ho L, Morisset J, Olson AL, Podolanczuk A, Poletti V, Selman M, Ewing T, Jones S, Knight SL, Ghazipura M, Wilson KC (2022) Idiopathic pulmonary fibrosis (an update) and progressive pulmonary fibrosis in adults: an official ATS/ERS/JRS/ALAT clinical practice guideline. Am J Respir Crit Care Med 205(9):e18–e47. 10.1164/rccm.202202-0399ST 35486072 10.1164/rccm.202202-0399ST PMC9851481

[2] Wijsenbeek M, Molina-Molina M, Chassany O, Fox J, Galvin L, Geissler K, Hammitt KM, Kreuter M, Moua T, O’Brien EC, Slagle AF, Krasnow A, Reaney M, Baldwin M, Male N, Rohr KB, Swigris J, Antoniou K (2022) Developing a conceptual model of symptoms and impacts in progressive fibrosing interstitial lung disease to evaluate patient-reported outcome measures. ERJ Open Res 8(2):00681–2021. 10.1183/23120541.00681-2021 35509443 10.1183/23120541.00681-2021 PMC9062300

[3] Takei R, Matsuda T, Fukihara J, Sasano H, Yamano Y, Yokoyama T, Kataoka K, Kimura T, Suzuki A, Furukawa T, Fukuoka J, Johkoh T, Kondoh Y (2023) Changes in patient-reported outcomes in patients with non-idiopathic pulmonary fibrosis fibrotic interstitial lung disease and progressive pulmonary fibrosis. Front Med (Lausanne) 10:1067149. 10.3389/fmed.2023.1067149 37457568 10.3389/fmed.2023.1067149 PMC10347395

[4] Green R, Baldwin M, Pooley N, Misso K, Mölken MPR, Patel N, Wijsenbeek MS (2024) The burden of cough in idiopathic pulmonary fibrosis and other interstitial lung diseases: a systematic evidence synthesis. Respir Res 25(1):325. 10.1186/s12931-024-02897-w 39192278 10.1186/s12931-024-02897-w PMC11351049

[5] Wijsenbeek M, Swigris JJ, Inoue Y, Kreuter M, Maher TM, Suda T, Baldwin M, Mueller H, Rohr KB, Flaherty KR, INBUILD Trial Investigators (2024) Effects of nintedanib on symptoms in patients with progressive pulmonary fibrosis. Eur Respir J 63(2):2300752. 10.1183/13993003.00752-2023 38135442 10.1183/13993003.00752-2023 PMC10831140

[6] Ryerson CJ, Arean PA, Berkeley J, Carrieri-Kohlman VL, Pantilat SZ, Landefeld CS, Collard HR (2012) Depression is a common and chronic comorbidity in patients with interstitial lung disease. Respirology 17(3):525–532. 10.1111/j.1440-1843.2011.02122.x 22221976 10.1111/j.1440-1843.2011.02122.x

[7] Yalnız E, Polat G, Demirci F, Deniz S, Karadeniz G, Aydınlı E, Vayisoglu G, Ayrancı A (2019) Are idiopathic pulmonary fibrosis patients more anxious and depressive than patients with other interstitial lung disease? Sarcoidosis Vasc Diffuse Lung Dis 36(4):294–301. 10.36141/svdld.v36i4.8418 32476965 10.36141/svdld.v36i4.8418 PMC7247093

[8] Algamdi M, Sadatsafavi M, Fisher JH, Morisset J, Johannson KA, Fell CD, Kolb M, Manganas H, Cox G, Gershon AS, Halayko AJ, Hambly N, Khalil N, Shapera S, To T, Wilcox PG, Guler S, Ryerson CJ (2019) Costs of workplace productivity loss in patients with fibrotic interstitial lung disease. Chest 156(5):887–895. 10.1016/j.chest.2019.04.016 31051170 10.1016/j.chest.2019.04.016

[9] Algamdi M, Sadatsafavi M, Fisher JH, Morisset J, Johannson KA, Fell CD, Kolb M, Manganas H, Cox G, Gershon AS, Halayko AJ, Hambly N, Khalil N, Shapera S, To T, Wilcox PG, Guler S, Ryerson CJ (2020) Costs of workplace productivity loss in patients with connective tissue disease-associated interstitial lung disease. Ann Am Thorac Soc 17(9):1077–1084. 10.1513/AnnalsATS.201911-822OC 32437249 10.1513/AnnalsATS.201911-822OC

[10] Knarborg M, Løkke A, Hilberg O, Ibsen R, Sikjaer MG (2022) Direct and indirect costs of systemic sclerosis and associated interstitial lung disease: a nationwide population-based cohort study. Respirology 27(5):341–349. 10.1111/resp.14234 35224821 10.1111/resp.14234 PMC9306585

[11] Lobo LJ, Liu Y, Li P, Ramaswamy M, Ramaswamy M, Swaminathan AC, Veeraraghavan S, Fan Y, Neely ML, Palmer SM, Olson AL, ILD-PRO Registry investigators (2024) Design and baseline characteristics of the ILD-PRO Registry in patients with progressive pulmonary fibrosis. BMC Pulm Med 24(1):468. 10.1186/s12890-024-03247-8 39334205 10.1186/s12890-024-03247-8 PMC11438290

[12] Reilly MC, Zbrozek AS, Dukes EM (1993) The validity and reproducibility of a work productivity and activity impairment instrument. PharmacoEconomics 4(5):353–365. 10.2165/00019053-199304050-00006 10146874 10.2165/00019053-199304050-00006

[13] Reilly MC (2004) Work Productivity and Activity Impairment Questionnaire: General Health V2.0 (WPAI:GH). http://www.reillyassociates.net/wpai_gh.html 10.1111/j.1524-4733.2006.00101.x 16689715

[14] U.S. Bureau of Labor Statistics Median usual weekly earnings of full-time wage and salary workers by age and sex. Accessed via: https://www.bls.gov/charts/usual-weekly-earnings/usual-weekly-earnings-current-quarter-by-age.htm. Accessed: 10 December 2024

[15] Crawford B, Monz B, Hohlfeld J, Roche N, Rubin B, Magnussen H, Nivens C, Ghafouri M, McDonald J, Tetzlaff K (2008) Development and validation of a cough and sputum assessment questionnaire. Respir Med 102(11):1545–1555. 10.1016/j.rmed.2008.06.009 18662868 10.1016/j.rmed.2008.06.009

[16] Jones PW, Quirk FH, Baveystock CM (1991) The St George’s Respiratory Questionnaire. Respir Med 85 Suppl B 25–31. 10.1016/s0954-6111(06)80166-6 10.1016/s0954-6111(06)80166-6 1759018

[17] Ley B, Ryerson CJ, Vittinghoff E, Ryu JH, Tomassetti S, Lee JS, Poletti V, Buccioli M, Elicker BM, Jones KD, King TE Jr, Collard HR (2012) A multidimensional index and staging system for idiopathic pulmonary fibrosis. Ann Intern Med 156(10):684–691. 10.7326/0003-4819-156-10-201205150-00004 22586007 10.7326/0003-4819-156-10-201205150-00004

[18] Swigris JJ, Brown KK, Behr J, du Bois RM, King TE, Raghu G, Wamboldt FS (2010) The SF-36 and SGRQ: validity and first look at minimum important differences in IPF. Respir Med 104(2):296–304. 10.1016/j.rmed.2009.09.006 19815403 10.1016/j.rmed.2009.09.006 PMC2856332

[19] Rebelo P, Oliveira A, Paixão C, Valente C, Andrade L, Marques A (2020) Minimal clinically important differences for patient-reported outcome measures of cough and sputum in patients with COPD. Int J Chron Obstruct Pulmon Dis 15:201–212. 10.2147/COPD.S219480 32099345 10.2147/COPD.S219480 PMC6996113

[20] Kigozi J, Jowett S, Lewis M, Barton P, Coast J (2017) The estimation and inclusion of presenteeism costs in applied economic evaluation: a systematic review. Value Health 20(3):496–506. 10.1016/j.jval.2016.12.006 28292496 10.1016/j.jval.2016.12.006

[21] Wuyts WA, Papiris S, Manali E, Kilpeläinen M, Davidsen JR, Miedema J, Robalo-Cordeiro C, Morais A, Artés M, Asijee G, Cendoya D, Soulard S (2020) The burden of progressive fibrosing interstitial lung disease: a DELPHI approach. Adv Ther 2020;37(7):3246–64. 10.1007/s12325-020-01384-0 10.1007/s12325-020-01384-0 PMC7467418 32445186

[22] Løkke A, Castello L, Pinheiro Martins P, Soulard S, Hilberg O (2023) Burden of disease and productivity loss in the European Economic Area in patients affected by fibrosing interstitial lung disease. Adv Ther 40(12):5502–5518. 10.1007/s12325-023-02701-z 37837527 10.1007/s12325-023-02701-z PMC10611590

[23] Olson AL, Hartmann N, Patnaik P, Garry EM, Bohn RL, Singer D, Baldwin M, Wallace L (2022) Healthcare resource utilization and related costs in chronic fibrosing interstitial lung diseases with a progressive phenotype: a US claims database analysis. Adv Ther 39(4):1794–1809. 10.1007/s12325-022-02066-9 35199282 10.1007/s12325-022-02066-9 PMC8990938

[24] Singer D, Bengtson LGS, Conoscenti CS, Anderson AJ, Brekke L, Shetty SS, Brown KK (2022) Burden of illness in progressive fibrosing interstitial lung disease. J Manag Care Spec Pharm 28(8):871–880. 10.18553/jmcp.2022.28.8.871 35876293 10.18553/jmcp.2022.28.8.871 PMC10373037

[25] Nili M, Steffens A, Anderson A, Brekke L, Grace Johnson M, Veeranki P, Olson AL (2024) Health care costs and utilization of progressive fibrosing lung disease by underlying interstitial lung disease type. J Manag Care Spec Pharm 30(2):163–174. 10.18553/jmcp.2024.30.2.163 38308627 10.18553/jmcp.2024.30.2.163 PMC10839459

[26] Swigris JJ, Stewart AL, Gould MK, Wilson SR (2005) Patients’ perspectives on how idiopathic pulmonary fibrosis affects the quality of their lives. Health Qual Life Outcomes 3:61. 10.1186/1477-7525-3-61 16212668 10.1186/1477-7525-3-61 PMC1276807

[27] Schoenheit G, Becattelli I, Cohen AH (2011) Living with idiopathic pulmonary fibrosis: an in-depth qualitative survey of European patients. Chron Respir Dis 8(4):225–231. 10.1177/1479972311416382 21856780 10.1177/1479972311416382

[28] Center for Drug Evaluation and Research (CDER), U.S. Food and Drug Administration (FDA) The Voice of the Patient. A series of reports from the U.S. Food and Drug Administration’s (FDA’s) Patient-Focused Drug Development Initiative. Idiopathic Pulmonary Fibrosis. Available at: https://www.fda.gov/files/about%20fda/published/The-Voice-of-the-Patient--Idiopathic-Pulmonary-Fibrosis.pdf. Accessed: 10 December 2024

[29] Amati F, Spagnolo P, Ryerson CJ, Oldham JM, Gramegna A, Stainer A, Mantero M, Sverzellati N, Lacedonia D, Richeldi L, Blasi F, Aliberti S (2023) Walking the path of treatable traits in interstitial lung diseases. Respir Res 24(1):251. 10.1186/s12931-023-02554-8 37872563 10.1186/s12931-023-02554-8 PMC10594881

[30] Hofman DE, Magrì T, Moor CC, van Beek FT, Gür Y, Ooijevaar J, Wijsenbeek MS, de Mul M (2024) Patient-centered care in pulmonary fibrosis: access, anticipate, and act. Respir Res 25:395. 10.1186/s12931-024-02997-7 39487454 10.1186/s12931-024-02997-7 PMC11531140

[31] Khor YH, Goh NSL, McDonald CF, Holland AE (2017) Oxygen therapy for interstitial lung disease. a mismatch between patient expectations and experiences. Ann Am Thorac Soc 14(6):888–895. 10.1513/AnnalsATS.201611-934OC 28267349 10.1513/AnnalsATS.201611-934OC

[32] Tikellis G, Hoffman M, Mellerick C, Burge AT, Holland AE (2023) Barriers to and facilitators of the use of oxygen therapy in people living with an interstitial lung disease: a systematic review of qualitative evidence. Eur Respir Rev 32(169):230066. 10.1183/16000617.0066-2023 37611946 10.1183/16000617.0066-2023 PMC10445108

