# Supplementary material for: Workplace Productivity Loss in Patients with Progressive Pulmonary Fibrosis: Data from the ILD-PRO Registry
Source: Lung. 2026 May 27;204(1):32. doi: 10.1007/s00408-026-00895-x (PMC13319162; doi:10.1007/s00408-026-00895-x)
Supplement: Supplementary file 1 — Supplementary Material 1 [file 408_2026_895_MOESM1_ESM.docx]

**Supplementary appendix**

**Table S1.** Characteristics at enrollment of patients who completed the WPAI questionnaire (responders), patients who had not completed the questionnaire but were alive at the time the data were extracted from the database (live non-responders) and patients who had died

|  | **Responders (n=597)** | **Live non-responders (n=242)** | **Died  (n=161)** |
| --- | --- | --- | --- |
| Female | 367 (61.5) | 147 (60.7) | 90 (55.9) |
| Age, years | 67 (58, 73) | 68 (57, 73) | 72 (64, 76) |
| Race |  |  |  |
| White | 441 (73.9) | 162 (66.9) | 129 (80.1) |
| Black/African-American | 101 (16.9) | 44 (18.2) | 17 (10.6) |
| Asian | 10 (1.7) | 11 (4.5) | 5 (3.1) |
| Other | 22 (3.7) | 14 (5.8) | 3 (1.9) |
| Not reported | 23 (3.9) | 11 (4.5) | 7 (4.3) |
| Ethnicity |  |  |  |
| Hispanic/Latino | 31 (5.2) | 20 (8.3) | 7 (4.3) |
| Not reported | 23 (3.9) | 20 (8.3) | 8 (5.0) |

Data are median (Q1, Q3) or n (%) of patients.

**Figure S1.** Correlation (rho) between age and workplace productivity loss


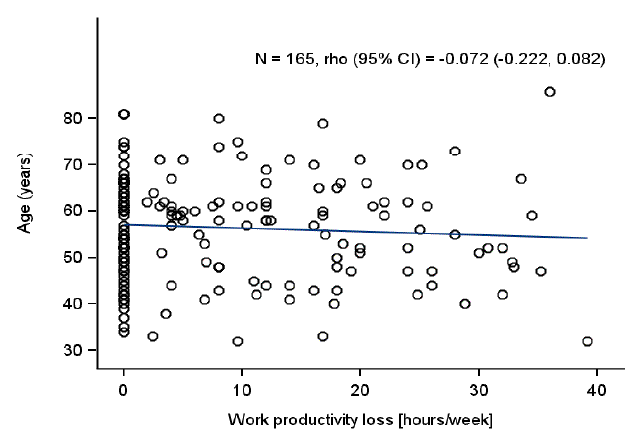


Excludes one patient with workplace productivity loss >80 hours/week.

**Figure S2.** Correlation (rho) between sex and workplace productivity loss


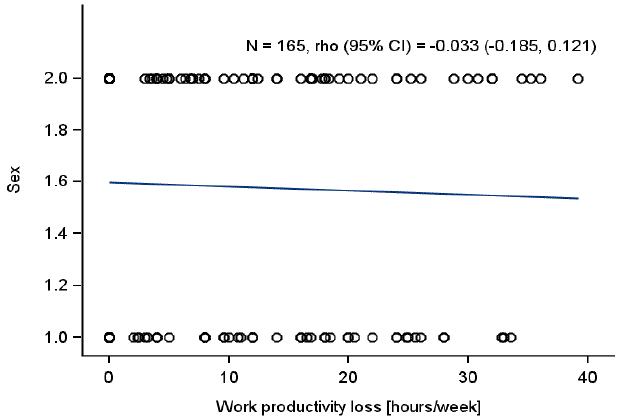


Excludes one patient with workplace productivity loss >80 hours/week.

**Figure S3.** Correlation (rho) between DLco % predicted and workplace productivity loss


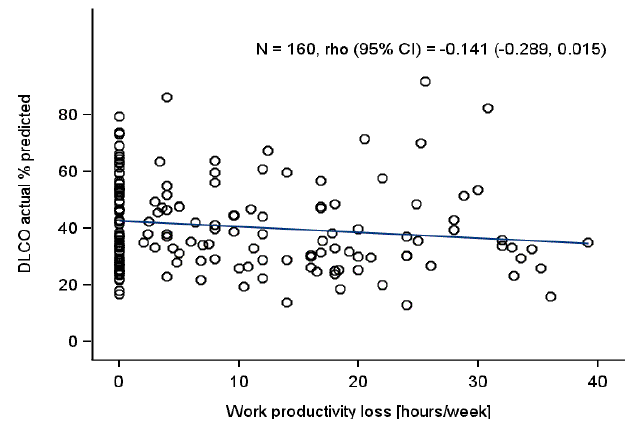


Excludes one patient with workplace productivity loss >80 hours/week.

DLco, diffusing capacity of the lungs for carbon monoxide.

**Figure S4.** Correlation (rho) between GAP index and workplace productivity loss


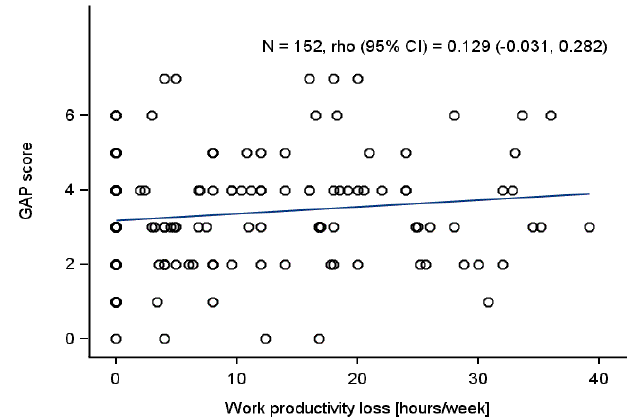


Excludes one patient with workplace productivity loss >80 hours/week.

GAP, gender, age, physiology.
